# Adjustable Rigid Interspinous Process Fixation: A Biomechanical Study of Segmental Lordosis and Interbody Loading in the Lumbar Spine

**DOI:** 10.7759/cureus.4317

**Published:** 2019-03-25

**Authors:** Anup Gandhi, Chris Ferry, Jason A Inzana, Steve W Chang, Ryan DenHaese

**Affiliations:** 1 Orthopaedics, Zimmer Biomet Spine, Westminster, USA; 2 Orthopaedics, Cooper Medical School of Rowan University, Camden, USA; 3 Orthopaedics, Telos Partners, Denver, USA; 4 Neurosurgery, Barrow Neurological Institute, St. Joseph's Hospital and Medical Center, Phoenix, USA; 5 Neurosurgery, AXIS Neurosurgery and Spine, Buffalo, USA

**Keywords:** interspinous process fixation, posterior fixation, lumbar, cadaveric, spine biomechanics, lateral lumbar interbody fusion, pedicle screw fixation, spine surgery, lordosis

## Abstract

Background

Rigid interspinous process fixation (ISPF) may serve as a minimally disruptive adjunct to lumbar interbody fusion. Previous biomechanical assessments of ISPF have demonstrated particularly advantageous outcomes in stabilizing the sagittal plane. However, ISPF has not been well characterized in regard to its impact on interbody load, which has implications for the risk of cage migration or subsidence, and sagittal alignment. The purpose of this study was to biomechanically assess in vitro the interbody load (IBL), focal lordosis (FL), and spinous process loading generated by in situ compression/distraction with a novel ISPF device capable of incremental in situ shortening/extension. Bilateral pedicle screw fixation (BPSF) was used as a control.

Methods

Two fresh frozen human lumbar spines were thawed and musculature was removed, leaving ligaments intact. Seven functional spinal units were iteratively tested, which involved a standard lateral discectomy, placement of a modified lateral cage possessing two load cells, and posterior fixation. BPSF and ISPF were performed at each level, with order of fixation was randomized. BPSF was first performed with maximum compressive exertion followed by 75% exertion to represent clinical application. The ISPF device was implanted at a neutral height and incrementally shortened/extended in situ in 1-mm increments. IBL and FL were measured under each condition. Loads on the spinous processes were estimated through bench-top mechanical calibration.

Results

No significant differences in IBL were observed, but the ISPF device produced a significantly greater change in FL compared to the clinically relevant BPSF compression. IBL, as a function of ISPF device height, expressed linear behavior during compression and exponential behavior during distraction.

Conclusions

The novel ISPF device produced clinically effective IBL and FL, performing well in comparison to BPSF. Additionally, incremental ISPF device manipulation demonstrated predictable and clinically safe trends regarding loading of the interbody space and spinous processes.

## Introduction

Rigid interspinous process fixation (ISPF) has been proposed as a less invasive alternative to pedicle screw fixation (PSF) for supplemental use in circumferential lumbar fusion [1-2]. While the body of literature evaluating the mechanical efficacy of ISPF continues to grow, particularly with respect to understanding segmental rigidity, the mechanisms through which sagittal correction and interbody (IB) loading are achieved and maintained with ISPF are not well characterized [3-10]. Given the previously demonstrated intraoperative benefits of ISPF, which include diminished bone invasion and limited midline paraspinal disruption, a greater biomechanical understanding of the ISPF technology would be highly advantageous in further defining its role as a minimally invasive adjunct in spine surgery [1-2,11-12].

With ISPF, compression or distraction is applied through the spinous processes and leverages a larger posterior moment arm about the IB space compared to manipulations through PSF. The larger moment arm achieved by ISPF may translate to substantial variations in the focal lordosis and IB loading, even with small adjustments. The option to apply compression or distraction in a controlled manner enables the surgeon to carefully modulate focal lordosis to achieve optimal sagittal balance. Simultaneously, the resulting compressive loading of the IB cage between the vertebral endplates may help resist migration, but should also be moderated to minimize the risk of subsidence [13-15]. Therefore, it is essential to characterize the focal lordosis and IB loading during spinous process manipulation via ISPF in comparison with the standard clinical technique of bilateral PSF (BPSF).

Accordingly, the objective of this study was to evaluate the translated effects of spinous process manipulation, via a novel ISPF device, on IB loading and sagittal correction. The novel ISPF device, which can be incrementally shortened/extended in situ, provides an ideal mechanism to achieve precise compression/distraction of the spinous processes without the need for device substitution or additional instrumentation. The IB loading and focal lordosis induced through the ISPF device was compared with BPSF - the gold standard for posterior fixation. In addition, the loads applied to the spinous processes were characterized to compare with previously measured failure loads.

## Materials and methods

Cadaveric specimen preparation

Two fresh-frozen human cadaveric spines were used in this study (age-sex: 57 years, male; 61 years, male). Each spine was thawed at room temperature and the lumbosacral specimens (L1-S1) were dissected out. Ligamentous structures were maintained. Residual musculature and adipose tissue were removed. Osseous structural integrity was confirmed via standard anteroposterior and lateral radiographs. Any specimens exhibiting previous lumbosacral surgery, excessive degeneration, or anatomical discrepancy were excluded. No structural failures or abnormalities were observed during testing.

**Figure 1:**
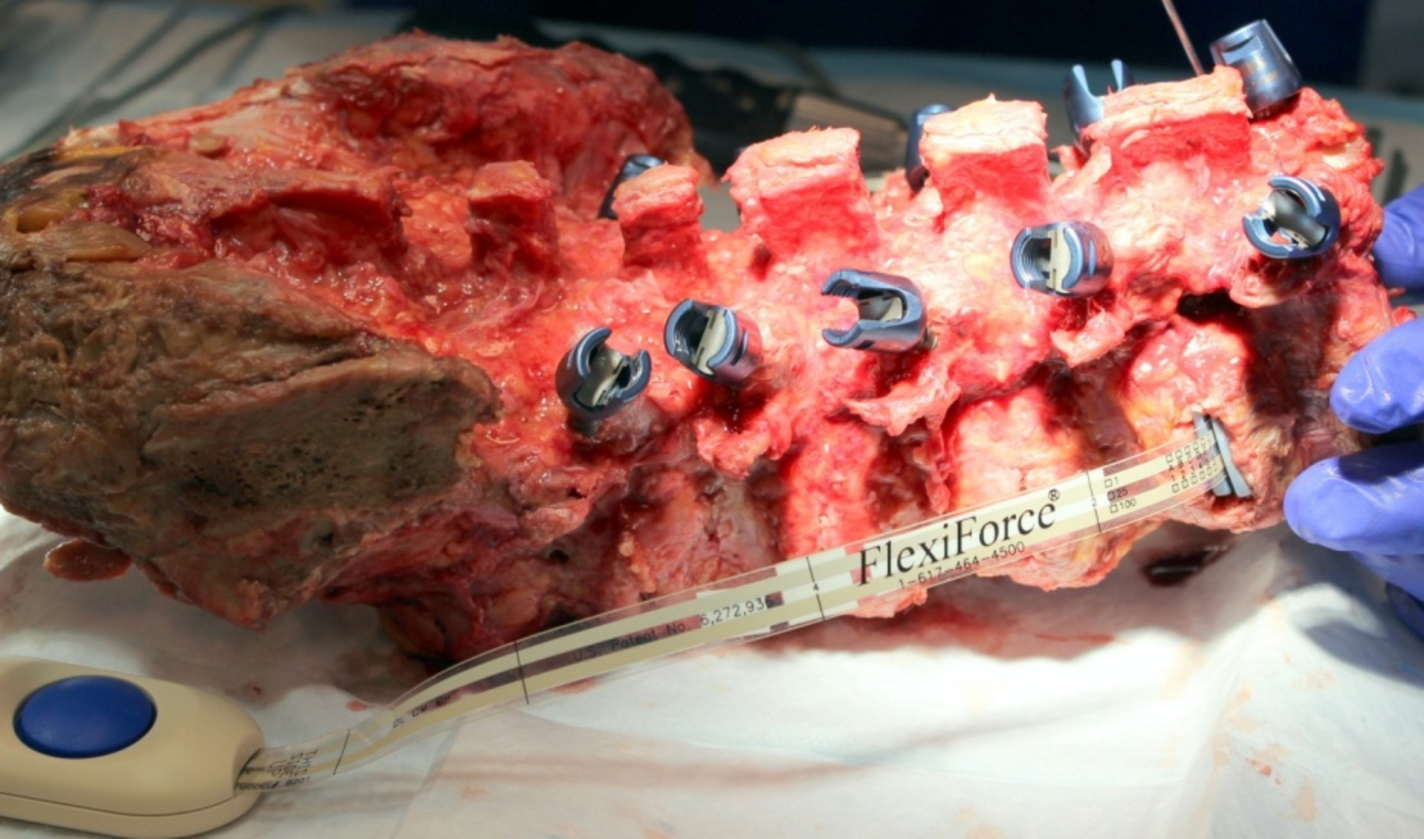
Test specimen with bilateral pedicle screw instrumentation Note that a modified, force-sensing lateral interbody cage has been inserted at the most superior level.

One level was excluded due to damage incurred at L1 during specimen dissection/preparation, leaving a combined total of seven (*n* = 7) functional spinal units (FSUs) that were utilized in the study. The specimens were preemptively instrumented with bilateral pedicle screws (Silverton® Spinal Fixation System; Zimmer Biomet Spine, Westminster, CO USA) at levels L1-L5 (Figure 1). Doing so ensured the consistent composition of the vertebral bodies throughout all testing and reduced the amount of manipulation to the vertebral column once testing had commenced. The pedicle screws did not contact any adjacent vertebrae or screws; hence, the IB loads and spinal motion were not affected by the preemptive screw insertion. Connecting rods were not inserted during this initial instrumentation phase. Prior to both ISPF and BPSF instrumentation, the interspinous and supraspinous ligaments were preemptively removed to accommodate the ISPF device. This does not alter the results of BPSF fixation, considering that distraction with BPSF was not measured in this study and those ligaments do not provide notable compressive resistance.

**Figure 2:**
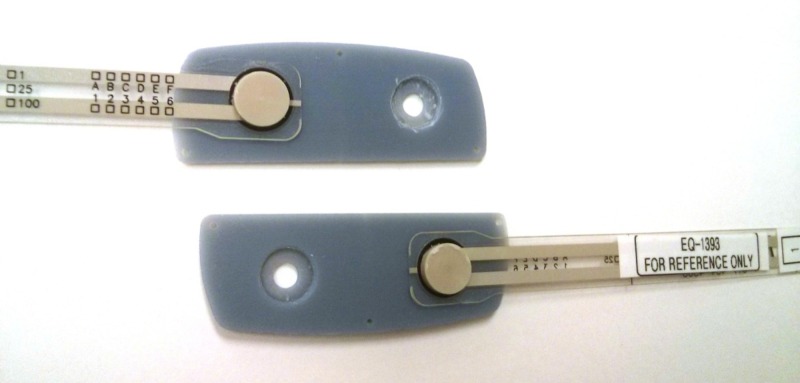
Modified, force-sensing lateral interbody cage Note the two load cells situated between the superior and inferior end-plates of the cage.

Following pedicle screw placement, a partial discectomy was performed, through a standard lateral approach to accommodate the placement of a modified lateral lumbar interbody fusion (LLIF) cage. The cage (22 mm (W) × 60 mm (L)) was modified such that it possessed two load cells (FlexiForce®; Tekscan Inc., Boston, MA USA; Figure 2). The load cells acted as a bridge between the inferior/superior endplates of the implant, and so all loading transduced through the cage was assumed by the load cells. The two load cell forces were summed to determine the total IB cage load at each treated level. The cage footprint, profile, and surface design were consistent with a commercially available lateral cage (Timberline® Lateral Fusion System; Zimmer Biomet Spine, Westminster, CO USA). An appropriate cage height was determined specific to each affected level and was accounted for in the thickness of the cage endplates. The sequence of testing was randomized such that four levels were tested with BPSF first and three with the ISPF device first.

BPSF testing

BPSF testing was performed in two stages at each treated level. Following preparation of the disc space, the modified lateral cage of an appropriate height was placed. The baseline IB cage load was recorded, and lateral fluoroscopic images were taken prior to each loading stage.

**Figure 3:**
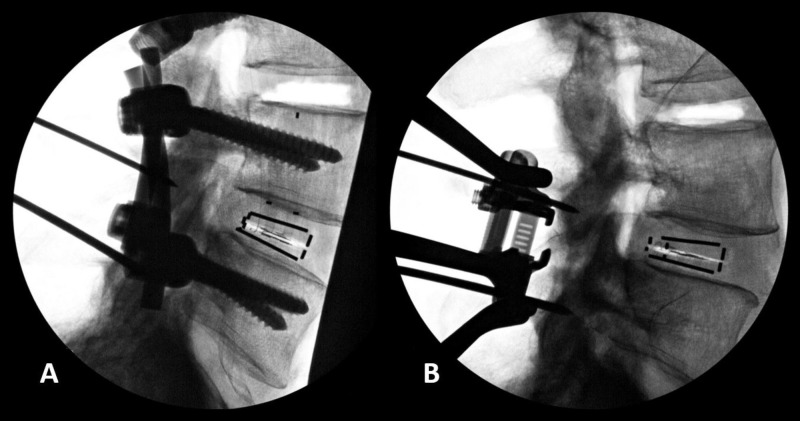
Lateral fluoroscopic images Bilateral pedicle screw fixation (A) and interspinous process fixation (B) constructs with the modified, force-sensing lateral cage placed at the index level.

Following baseline parameter measurement, bilateral connecting rods were fixed to the pedicle screws across a single FSU at a time under maximum attainable compression. The maximum compressive force was recorded using a fixed load cell on the compressor handles. Final set screw tightening was then performed and the IB cage load was recorded and lateral fluoroscopic images were taken. Next, the set screws were loosened from the connecting rods and the vertebral segment was allowed to relax to a neutral state. The pedicle screws were then recompressed to 75% of the previously measured maximum compressive force, which was determined by the surgeon authors to be representative of the exertion during clinical application. Since the force applied by each surgeon may be subjective and variable, the maximum compression was applied by a single individual to minimize inter-specimen variability and the load values were measured to ensure consistency. The set screws were then retightened to secure the connecting rods and the IB cage load and the lateral fluoroscopic images were collected (Figure 3A). The set screws and connecting rods were then removed and the vertebral segment was allowed to return to a neutral state.

ISPF testing

**Figure 4:**
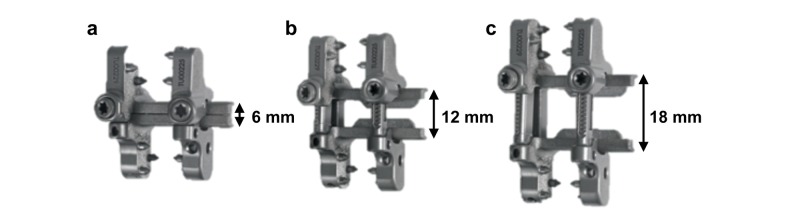
Novel adjustable interspinous process fixation device Interspinous process fixation device demonstrating various post heights for maximal compression (A) and maximal distraction (C) relative to the center height (B). Image source: Zimmer Biomet Spine, Westminister, CO USA

**Figure 5:**
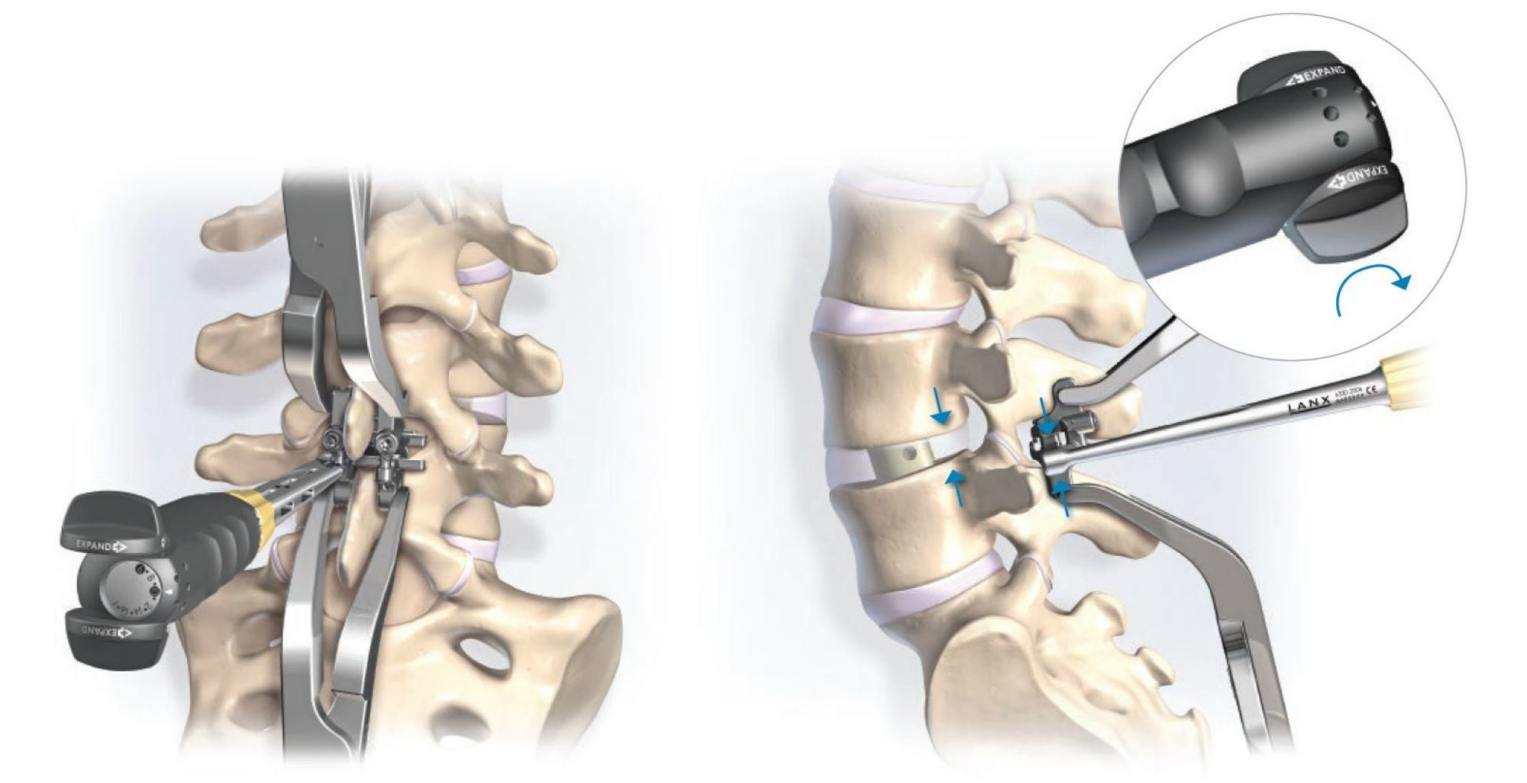
Schematic representation of the adjuster tool Note the schematic (right) demonstrates device compression; however, expansion occurs through the same mechanism by reversing the dial. Image source: Zimmer Biomet Spine, Westminister, CO USA

The ISPF device (Alpine XC^TM^ Adjustable Fusion System; Zimmer Biomet Spine, Westminster, CO USA) was implanted with a device post height of 14 mm (Figures 4, 5). The post is considered the adjustable (axial) portion of the device that sits within the interspinous space when the device is implanted. To maintain consistency between specimens, the spinous processes were trimmed when necessary to ensure that a post height of 14 mm was consistently an appropriate fit for the neutral loading state. During clinical application, trimming of the spinous process is not necessary considering that the post height can be adjusted to fit each patient and level. Sagittal compression of the spinous processes by the ISPF device was achieved such that the device spikes were seated with good bone apposition. Additionally, device placement was as far anterior and as close to the laminar junction as possible. Once in place, the post height of the device can be compressed downward from 14 mm to 6 mm or expanded upward from 14 mm to 18 mm.

Following device fixation to the spinous processes, the device post height was decreased in 1-mm increments to the minimum post height of 6 mm. This 8 mm of compression was determined by the surgeon authors to be clinically appropriate for both specimens given their respective anatomy. However, the authors emphasize that 8 mm of compression is not to be considered a standard value and that the degree of compression or distraction is dependent upon patient specific anatomy.

IB cage load measurements were collected at each 1-mm increment during compression. The device was then expanded to its maximum post height of 18 mm. Similarly, IB cage load was recorded at each 1-mm increment. The torque applied to the height adjustment instrument was also recorded at each 1 mm increment during compression and distraction. Torque values were used in subsequent calculations to estimate the force exerted by the ISPF device on the spinous processes. Lateral fluoroscopic images were collected at neutral, fully compressed, and fully distracted states (Figure 3B). Subsequent measurement of focal lordosis for both fixation types was performed using Cobb’s method. 

Spinous process force calculation

**Figure 6:**
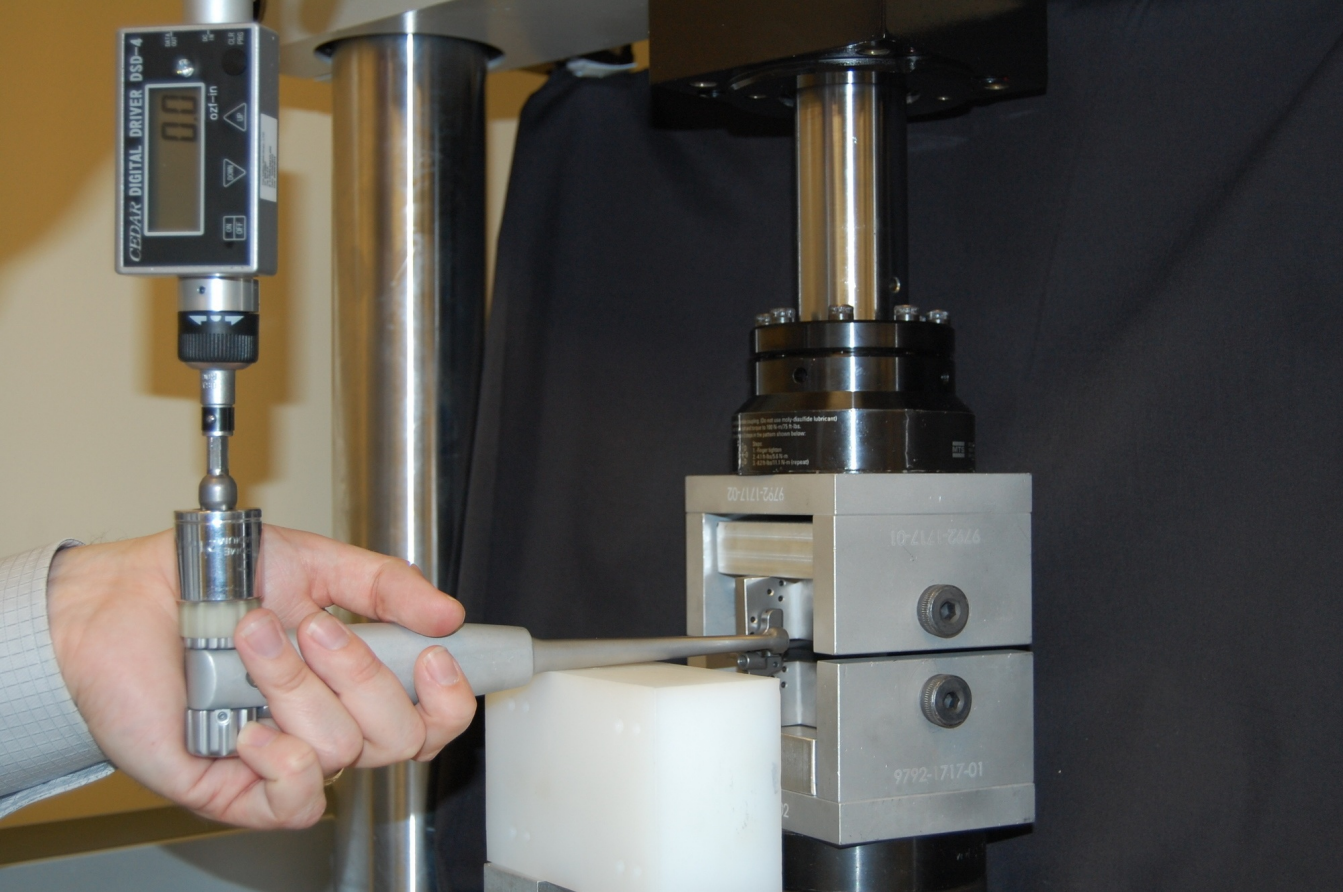
Test assembly to determine device force as the function of height Note the interspinous process fixation device assembled to two modified ASTM (American Society for Testing and Materials) F1717 stainless steel test blocks, within a load cell testing frame, with adjuster tool attached.

Independent of the cadaveric testing phase, a calibration plot was developed to estimate the forces applied by the ISPF device on the spinous processes while in compression/distraction. The ISPF device was first assembled to two modified ASTM (American Society for Testing and Materials) F1717 stainless steel test blocks and then placed within a test frame possessing a load cell (MTS Bionix® Tabletop Test Systems; MTS Systems Corporation, Eden Praire, MN USA; Figure 6). The ISPF device was then compressed by applying known torque values to the adjuster tool. The corresponding force transmitted by the ISPF device on the test blocks was measured by the load cell. The applied torque values were plotted against their corresponding loads. A best-fit linear equation was then fit to the data. This linear standard curve was utilized to convert the torque values that were measured during cadaveric testing to the estimated forces exerted by the ISPF device on the spinous processes.

Statistical methods

The change (%) in IB load, relative to baseline, and change in focal lordosis (deg.) were compared between the ISPF device at its in situ compressed state (post height = 6 mm), the ISPF device at its in situ distracted state (post height =18 mm), BPSF under 75% exertion, and BPSF under 100% exertion by Friedman’s test (non-parametric repeated measures ANOVA) with Dunn’s test for multiple post hoc comparisons. Multiplicity-adjusted *p*-values less than 0.05 were considered statistically significant. Non-parametric tests were used for statistical testing due to the small sample size. To ensure consistency between the specimens, the BPSF compression loads were checked for outliers using the robust regression and outlier removal (ROUT) method with a Q coefficient equal to 5%. All statistical analyses were performed using GraphPad Prism 7.01 (GraphPad Software, San Diego, CA USA).

## Results

BPSF compression

The maximum load (100% exertion) applied to the instrument handles during BPSF compression was 204 ± 8 N (range: 194-218 N) and the clinically relevant loading (75% of maximum) was 157 ± 11 N (range: 148-178 N). There were zero statistical outliers according to the ROUT method with a Q coefficient equal to 5%, suggesting good inter-specimen repeatability.

Interbody loading

**Figure 7:**
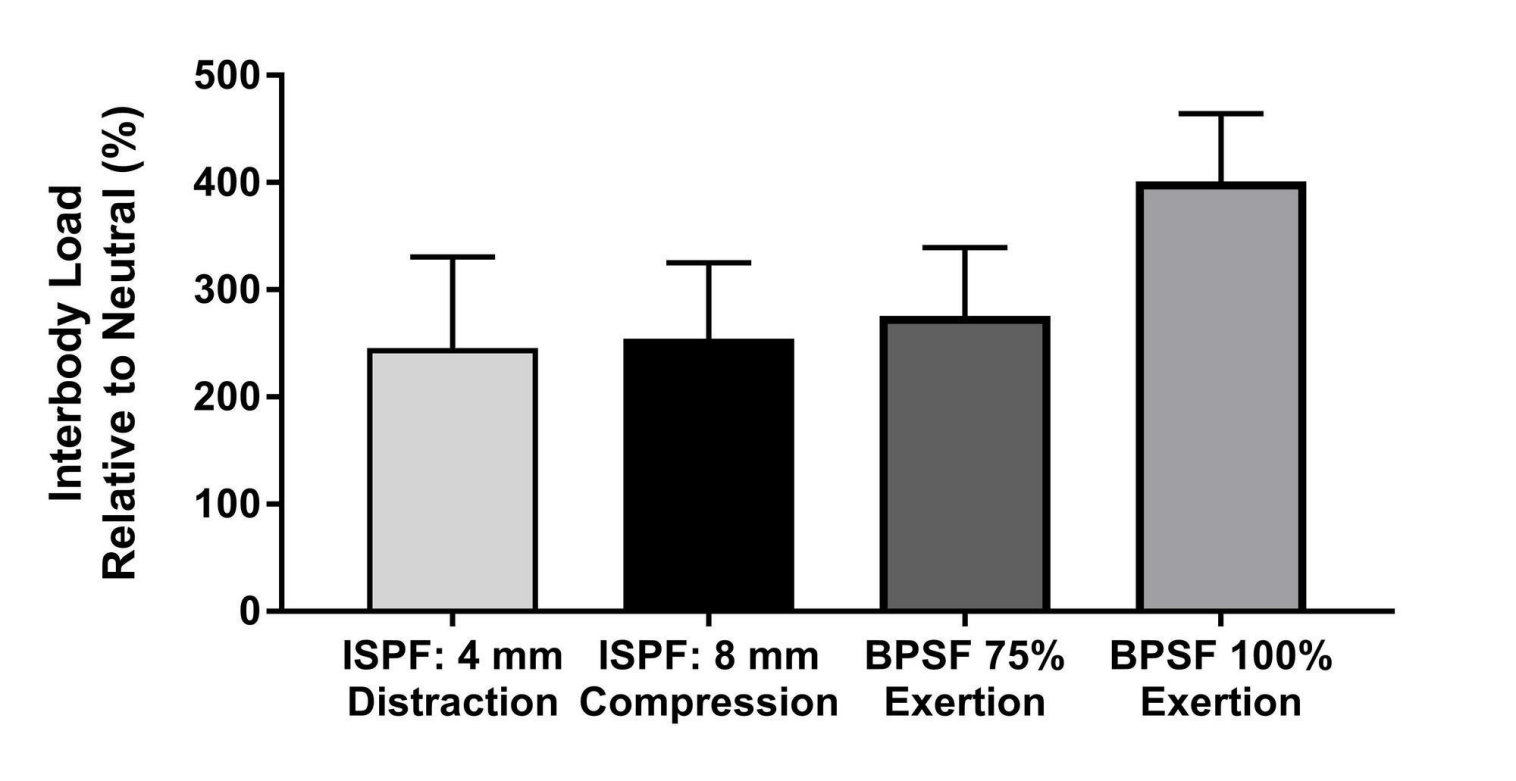
Interbody load versus fixation condition No significant differences in the interbody load, relative to neutral, were observed between the four posterior fixation/manipulation techniques. Note that bilateral pedicle screw fixation under the extreme case of 100% compressive exertion tended to produce the greatest load relative to bilateral pedicle screw fixation at 75% exertion (clinically relevant loading) and the interspinous process fixation techniques (*p* = 0.068). Values represent the mean and error bars are standard error measurements. ISPF, interspinous process fixation; BPSF, bilateral pedicle screw fixation.

**Figure 8:**
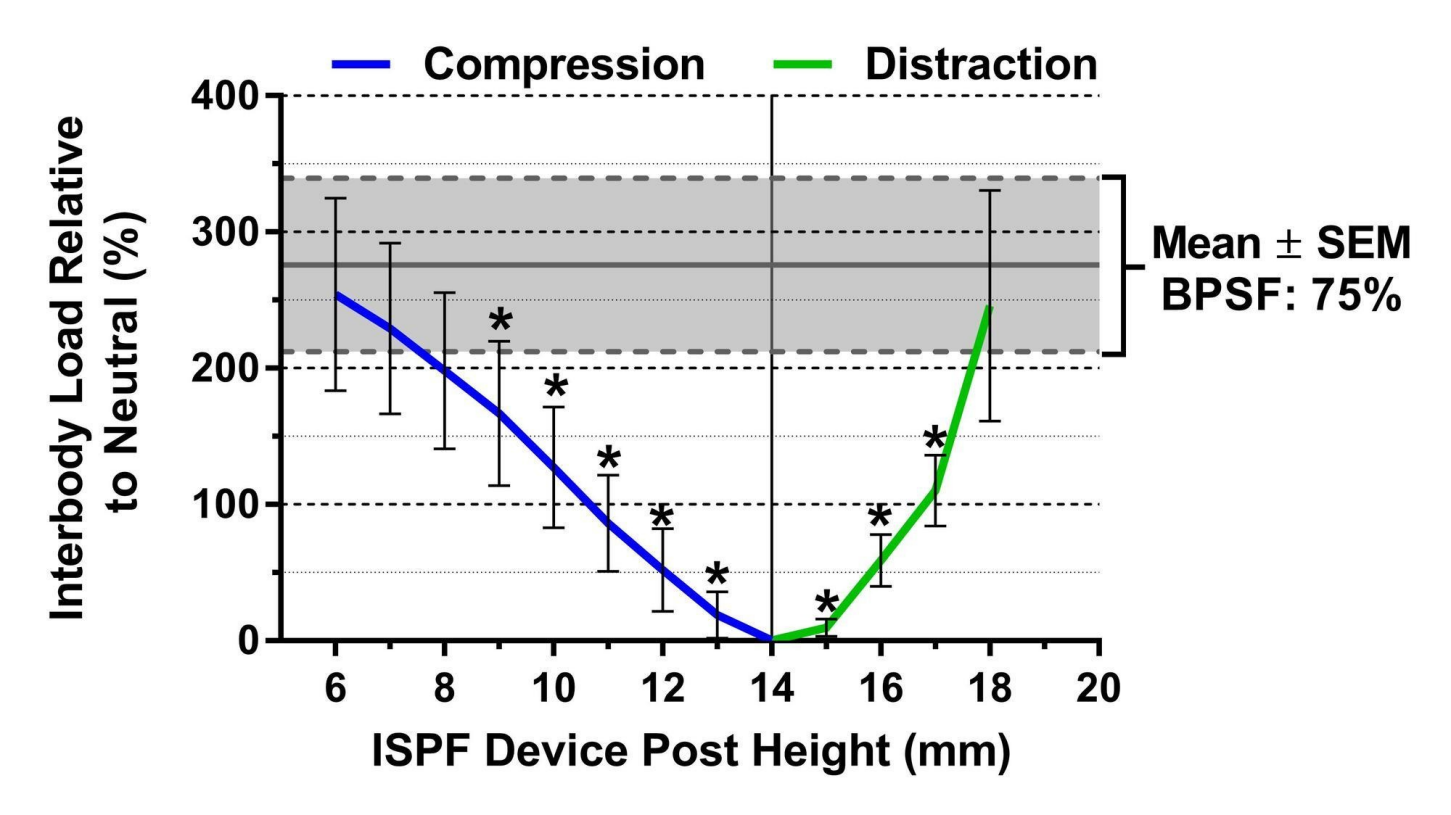
Interbody load versus interspinous fixation device post height The compressive interbody cage load increased regardless of whether the interspinous process fixation device is compressed or distracted. During compression, the interbody cage load tends to increase linearly, while distraction produced second-order or exponential increases in interbody cage load. * denotes*p* <0.05 vs. bilateral pedicle screw fixation at 75% exertion by Friedman’s test, followed by Dunn’s test for multiple post hoc comparisons. Lines represent the mean and error bars are standard error measurements. BPSF, bilateral pedicle screw fixation; ISPF, interspinous process fixation; SEM, standard error measurement

No significant differences in IB load relative to baseline (%) were observed between the four testing conditions. ISPF in maximal compression (6 mm) or distraction (18 mm) and the clinically relevant BPSF compression produced similar IB load values (Figure 7). Applying maximum compression on the BPSF construct tended to produce the greatest IB loads, but were not significantly different from the other 3 test conditions (p = 0.068). The difference in IB load was significantly different between BPSF at 75% effort and the ISPF device when it was adjusted less than 6 mm in compression or 4 mm in distraction (Figure 8). The mean compressive IB load ranged from 101 to 329 N over the four test conditions.

The mean IB load as a function of ISPF device post height demonstrated linear behavior (*R*^2^= 0.995) in the compression phase, and non-linear, second-order behavior in distraction (*R*^2 ^= 0.998; Figure 8).

Segmental lordosis

**Figure 9:**
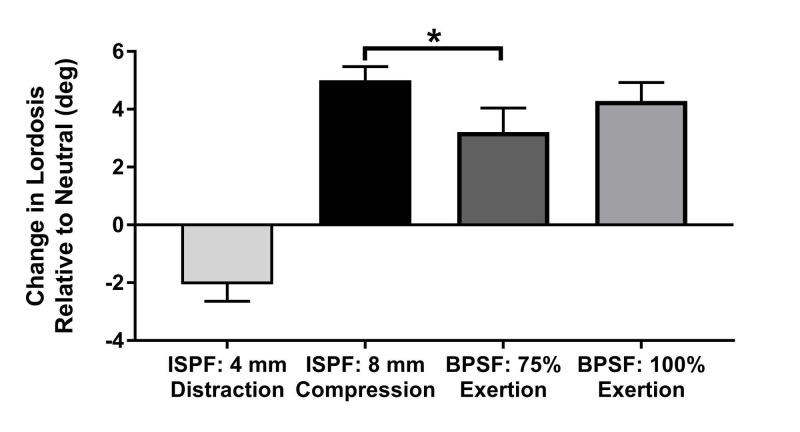
Change in lordosis versus fixation condition Interspinous process fixation at 8 mm of compression produces significantly greater focal lordosis compared to clinically relevant compression with bilateral pedicle screw fixation. Note that the distraction of the interspinous process fixation successfully reduces focal lordosis. Values represent the mean and error bars are SEM. * denotes *p* <0.05 by Friedman’s test followed by Dunn’s test for multiple post hoc comparisons. Interspinous process fixation under distraction was not included in the statistical analysis due to the inverse nature of that technique. BPSF, bilateral pedicle screw fixation; ISPF, interspinous process fixation

Both BPSF test conditions, as well as the compressed ISPF device, increased the focal lordosis. To be expected, distraction with the ISPF device decreased the focal lordosis (Figure 9). The change in focal lordosis achieved with the compressed ISPF device was significantly greater than BPSF with 75% compression (5.0^o^ vs. 3.2^o^; *p* = 0.046). Distraction with the ISPF device was not included in the statistical comparisons due to the inversed result (decreased lordosis) with this method.

Spinous process loading

**Figure 10:**
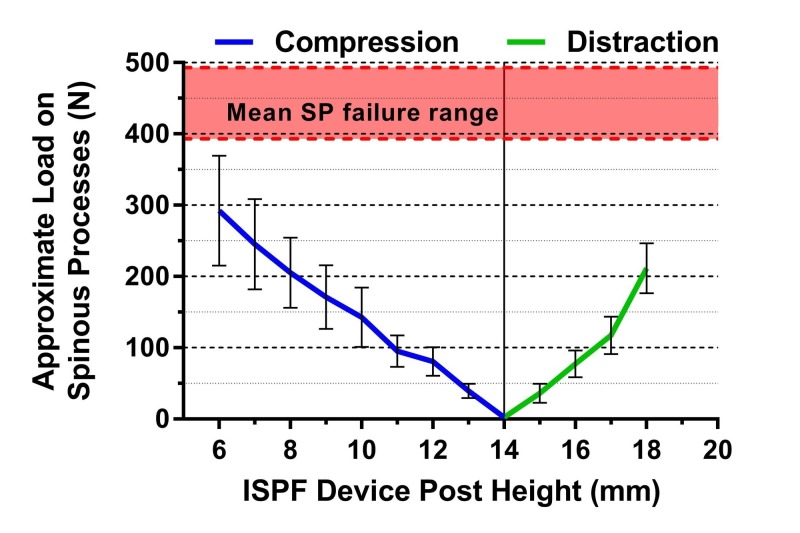
Approximate load on spinous processes versus interspinous fixation device post height A post height of 14 mm was the neutral height used during device insertion. Lines represent the mean and error bars are standard error measurements. Note that no fractures were observed in this study and that the estimated forces on the spinous processes are below the range of mean failure values reported in the literature [16]. SP, spinous process; ISPF, interspinous process fixation

The average estimated load applied to the spinous processes by the ISPF device when compressed to a post height of 6 mm was 292 N. When the ISPF device was fully expanded to 18 mm, the average estimated load on the spinous processes was 212 N (Figure 10). None of the spinous processes or the bone-device interfaces failed during testing.

## Discussion

Biomechanical outcomes

While considerable literature exists characterizing the biomechanical effectiveness of various posterior lumbar fixation constructs, the focus has almost exclusively been segmental rigidity/range-of-motion (ROM). IB loading and focal lordosis are rarely assessed in a cadaveric model [17-19]. However, given the continued incidence of cage subsidence and cage migration at noteworthy rates, improved understanding of such parameters is warranted [20-24]. When evaluating rigid ISPF, it is critical to consider IB loading and focal lordosis since the ISPF device leverages a larger moment arm compared to traditional BPSF, which may translate to greater biomechanical effects within the IB space. Indeed, significantly greater focal lordosis was observed during compression with the ISPF device compared to the clinically relevant BPSF compression, yet the compressive IB loads were similar.

The ideal compressive loading on an IB cage should be sufficient to prevent migration or expulsion, yet low enough to mitigate the risk of subsidence. Of course, the relevant load for each of these cases largely depends on the areal profile in contact with the vertebral endplates and is therefore specific to individual cage designs. Kwon and colleagues compressed a lateral cage footprint (18 mm × 60 mm) into vertebral endplates following cage insertion and observed compressive strength values of 1764 ± 966 N, which far exceed the IB loads observed herein [22]. On the other hand, resistance to migration or expulsion is more complicated and depends largely on the static friction coefficient between the cage and endplates, the stability of the spinal motion segment, as well as the areal contact. The IB loading induced by the clinically relevant levels of BPSF compression and ISPF compression or distraction were similar in this study, which suggests a similar level of resistance to migration in the neutral state. Although IB loading was not measured during kinematic analyses herein, previous studies have demonstrated similar rigidity between ISPF and BPSF in flexion-extension, but ISPF does tend to be less rigid in lateral bending and axial rotation [3,6]. Future studies that investigate the IB load during kinematic analysis may be useful to better understand the implications of supplemental ISPF or PSF for the risk of potential cage migration.

In contrast to an interspinous spacer, which acts more passively as a blocking device, the ISPF device can be utilized to actively increase focal lordosis through compression or focal kyphosis through distraction. Additionally, distraction can serve to relieve facet loads and open the spinal canal and neural foramens [25-27]. Interestingly, the distraction of the spinous processes using the ISPF device not only increased the compressive IB load but did so in exponential fashion. A 245% increase was achieved after just 4 mm of distraction, which was similar to that achieved with 8 mm of compression. This finding underscores the importance of the fulcrum that is leveraged during compression versus distraction of the posterior elements and has important implications for the effects on the anterior column. During ISPF compression, the IB cage itself likely served as the fulcrum considering that IB cages typically provide some distraction to the disc space and an increased compressive IB load was observed. In contrast, the effects on the anterior column from spinous process distraction are likely influenced by tensioning structures, such as the posterior longitudinal ligament, which act as a middle column tensile fulcrum to increase the anterior compressive load [27]. This observation of increased load at the anterior column following posterior distraction is consistent with a previous study by Zheng et al, where the pressure distribution was mapped across the disc space [27].

In regard to sagittal correction, compression of the ISPF device (8 mm in situ) produced a significantly greater change in focal lordosis compared with BPSF under 75% compression. Furthermore, distraction with the ISPF device (4 mm in situ) produced an increase in local kyphosis of 2.0^o^, demonstrating an effective range of sagittal correction of 7.0^o^ (compressed-to-distracted states). Such range, achieved in a calculable manor, allows the surgeon to consider optimal sagittal balance while not compromising the load placed on the IB cage. This robust ability to manipulate the sagittal plane is consistent with previous ROM studies that have demonstrated an inherent ability of ISPF to resist flexion-extension [3-10].

A final important consideration of this study was whether or not the novel ISPF device exerts excessive forces on the spinous processes during compression or distraction. The estimated mean load on the spinous processes was 292 N under 8 mm of ISPF device compression and 212 N under 4 mm of distraction. Although the failure strength depends strongly on the bone size and quality of each individual, the applied loads were well below the range of mean failure strength values from previous studies (339-493 N) [16, 28]. Accordingly, no spinous process fractures were observed during this study. It is also important to reiterate that clinical application of this device requires appropriate selections of post height and compression/distraction for each patient to mitigate any risk of spinous process failure.

In this study, a novel ISPF device, which affords incremental compression/distraction in situ, was compared with traditional BPSF under varied degrees of compression. While the semi-quantitative approach taken with BPSF was an inherent limitation to this study, it is consistent with the subjective nature of PSF in general. Compression/distraction with PSF is almost always achieved by surgeon feel alone, making calculable manipulation and characterization extremely challenging. Although this technique is naturally subjective, it is an important parameter to quantitatively evaluate in controlled studies, considering its potential impact on multiple outcomes. Therefore, the loads applied during BPSF compression were recorded to ensure repeatability at the maximal compressive effort and at a more clinically relevant level of compression (75% effort). The 75% effort level was deemed by the surgeon authors to be representative of clinical application. While the ISPF device can be precisely adjusted during compression/distraction, the extent of manipulation is still a subjective choice that depends on patient anatomy. In this study, 8 mm of compression (reduction in post height from 14 mm to 6 mm) was deemed to be clinically appropriate for the specific anatomies. However, since IB load was measured at 1 mm increments, additional comparisons with BPSF at 75% compression can be made (Figure 8). Further comparisons with BPSF at 100% effort were not made, considering such extreme compression is not reflective of clinical scenarios and is often avoided, as excessive stress at the screw-to-bone interface and at the rod-to-set screw juncture predisposes the screw to pull-out and construct failure [29].

Clinical implications

While the amount of evidence regarding rigid ISPF is still limited, early clinical and biomechanical assessments have demonstrated good utility, particularly in anterior lumbar interbody fusion (ALIF) and LLIF application [1-12]. Given the ability for anterior and lateral cages alone to provide significant reduction and stability, notably in the axial and coronal planes, extensive and invasive posterior fixation may be replaced by less invasive supplemental fixation strategies in many cases [3-4, 6-7]. ISPF is a minimally invasive strategy that provides a robust mechanism for locking the sagittal plane while largely preserving the midline structures, making it a particularly well-suited adjunct to a large anterior or lateral IB cage.

Despite such an influential stabilization mechanism with ISPF, the ability to manipulate the spinous processes has traditionally been a challenge. Drilling and pinning of the spinous processes have been the predominant technique to achieve the leverage required to compress the spinous processes and provide focal sagittal correction. Such a technique not only places an additional burden on the surgeon but can also predispose the spinous processes to the fracturing. Furthermore, similar to PSF, the technique is entirely subjective. No opportunity exists for precise, controlled manipulation. The novel adjustable ISPF device explored in this study may provide a solution to the challenges of this intervention. The in situ manipulation capabilities of the device avoid drilling and pinning while enabling incremental adjustments without the need for device substitution or repositioning. Additionally, the ability to compress or distract the segment post-implantation allows for a more favorable loading environment of the IB space.

Limitations

The authors acknowledge several inherent limitations of this study. Of previous note, the BPSF compression technique was somewhat subjective, but the protocol provided a reproducible mechanism through which consistent inter-specimen testing may be performed. Likewise, the 8 mm of compression with the ISPF device is a subjective surgeon choice that must be matched to the anatomy as best as possible. The extent to which the spinous processes can be manipulated without predisposing the bone mass to the fracturing is largely dependent upon patient anatomy and bone quality. The specimens utilized in this study were selected and, in some cases, the spinous processes were trimmed slightly to ensure consistency in the neutral post height and range of allowable compression/distraction. 

The authors also acknowledge the limitation in performing several iterative tests on each motion segment. This limitation requires the assumption that each test iteration is independent of any previous testing condition, which may not be accurate considering the viscoelastic nature of the soft tissue structures. However, this an inherent limitation of any biomechanical assessment of spinal motion segments in which iterative testing is performed at the same level. The use of a standardized protocol that included specimen relaxation, a randomized testing order, and outcomes normalized to baseline values was employed to mitigate this limitation as much as possible.

## Conclusions

The novel ISPF device demonstrated IB loading that is similar to that of BPSF under clinically relevant compression. Additionally, the ISPF device produced a greater increase in focal lordosis than BPSF, demonstrating the mechanical advantage of ISPF to readily provide sagittal correction through the extended lever arm of the spinous process. The ISPF device also demonstrated an ability to produce compressive loading of the IB space during distraction, resulting in increased focal kyphosis. Such a phenomenon shows that the novel ISPF device can afford a range of sagittal angulation that does not compromise IB loading. Given the less invasive nature and technical feasibility of the ISPF approach, such characteristics may present the novel ISPF device as a viable alternative to PSF in circumferential lumbar fusion.
